# Supplementary material for: Integrating Computational and Experimental Methods for the Rational Ecodesign and Synthesis of Functionalized Safe and Sustainable Biobased Oligoesters
Source: Polymers (Basel). 2025 Sep 19;17(18):2537. doi: 10.3390/polym17182537 (PMC12473331; doi:10.3390/polym17182537)
Supplement: Supplementary file 1 [file polymers-17-02537-s001.zip › new_ESI_polymers-3845901_.pdf]

Electronic Supplementary Information - ESI

Integrating computational and experimental methods for the rational ecodesign and synthesis of functionalized safe and sustainable biobased oligoesters

Federico Zappaterra, Anamaria Todea, Fioretta Asaro, Pasquale Ditalia, Chiara Danielli, Monia Renzi, Serena Anselmi, Lucia Gardossi.

Raw data of the computational analysis

Tables S1A-L: Molecular descriptors calculated by VolSurf+ for trimers of the investigated monomers. Columns report: size and shape descriptors (V, S, R, G, Flex); hydrophilic and hydrophobic interaction volumes and capacity factors (W1–W8, CW1–CW8, D1–D8, CD1–CD8, DD1–DD8); INTEGY moments (IW1–IW4, ID1–ID4); H-bond donor/acceptor volumes (WO1–WO6, WN1–WN6); mixed descriptors (HL1–HL2, A, CP, POL, DIFF, MW, LOGP, LogD, PSA/HSA); charge state descriptors (NCC, AUS7.4, %FU4–%FU10); pharmacophoric triplets (DRDRDR–DODODO); and ADME-related parameters (SOLY, LgS3–LgS11, L0lgS–L4lgS, CACO2, SKIN, PB, LgBB, MetStab, VD, HTSFlag)

A. Objects	V	S	R	G	W1	W2	W3	W4	W5	W6	W7	W8
AA	362,375	283,788	1,27692	1,24053	940	797,25	533	278,625	153,875	86,5	39,375	7,25
BDO	256,875	215,72	1,19078	1,15981	702,375	597,125	353,125	165,75	89	42,375	6,75	0
GLY	227,25	193,407	1,17498	1,11296	718,125	635,875	393,75	202,25	111,125	56,375	18,375	3,75
ITA	306,75	246,757	1,24312	1,18821	833,75	707,5	474	259,75	153,25	85,25	42,5	10,625
MEG	173,25	158,788	1,09108	1,08599	606,75	558,875	332,625	160,875	84,5	39,375	4,5	0
PDO	214,875	185,358	1,15924	1,10435	654,5	569,25	341,125	168,75	90,25	42,25	6,5	0
BDOITA	482,875	365,74	1,32027	1,36206	1007,75	802,5	513,125	265	144,75	77,625	30,125	6,625
GLYITA	446,375	339,108	1,31632	1,31547	1113,25	970,5	652,5	341,25	189,875	101,875	41,875	7,125
MEGITA	390	298,38	1,30706	1,24269	999,75	861,625	536	254,625	132	65,25	19,625	3,875
PDOITA	435	325,149	1,33785	1,26738	1045,12	898,875	599,75	295,75	156,125	81,625	31,75	3,875
AABDOITA	753,25	547,467	1,37588	1,59909	1457,88	1152,75	766,25	391	213,875	118,875	54,75	10,25
AAGlyITA	710,375	507,934	1,39856	1,51529	1434,12	1212,25	857,25	480,875	273,625	149,25	66,625	13,5
AAMEGITA	672,125	490,885	1,36921	1,52157	1446,62	1214,62	821,25	419,25	219,75	115,25	55	10,625
AAPDOITA	709,75	507,805	1,39768	1,51605	1302,38	1043	730,5	389,75	218,5	121,25	57,875	12,25

B. Objects	D1	D2	D3	D4	D5	D6	D7	D8
AA	3,375	0	0	0	0	0	0	0
BDO	10,5	3,375	0,25	0	0	0	0	0
GLY	7,875	2,875	0,5	0	0	0	0	0
ITA	20,375	1,75	0,125	0	0	0	0	0
MEG	0	0	0	0	0	0	0	0
PDO	7,875	2,5	0,125	0	0	0	0	0
BDOITA	51,875	15	3,75	0,625	0,25	0	0	0
GLYITA	14,625	1,625	0,625	0	0	0	0	0
MEGITA	23	8,875	4,25	1,375	0,125	0	0	0
PDOITA	20,5	5,5	1,375	0,125	0	0	0	0
AABDOITA	68,75	22,25	4,25	0,75	0,25	0	0	0
AAGlyITA	50,125	11,75	2,5	0,625	0	0	0	0
AAMEGITA	30,25	9,625	5	2	0,125	0	0	0
AAPDOITA	56,625	17	4,125	1,25	0	0	0	0

C. Objects	WO1	WO2	WO3	WO4	WO5	WO6	WN1	WN2	WN3	WN4	WN5	WN6
AA	0	0	0	0	0	0	578,75	305,75	171,125	87,375	33,875	4,375
BDO	250,75	105,125	45,5	9	0	0	435,75	203,5	108,25	48,375	3,875	0
GLY	299,25	136,375	62,625	20,25	2,25	0	459,5	237,625	130,375	58,75	5,5	0
ITA	0	0	0	0	0	0	508,875	280	162,125	88,875	37,625	5,25
MEG	254,5	109	47,25	11,625	0	0	408,625	198	106,875	43,125	2	0
PDO	250,625	108,5	46,25	10,5	0	0	407,25	202,75	104,25	47,5	2,625	0
BDOITA	121,375	51,875	22,875	4,625	0	0	579,875	297,25	161,125	85,25	25,625	3,125
GLYITA	191,875	85,875	41,5	13,125	0,875	0	727,875	387,875	210,75	100,875	29,75	2,75
MEGITA	215,875	87,75	36,625	9,5	0	0	615,25	293,5	148,5	70,375	12,625	0,125
PDOITA	123,125	53,25	23,5	6,375	0	0	681,75	342,875	180,125	86,5	28	2,5
AABDOITA	0	0	0	0	0	0	836,375	429,375	233,125	121,625	47,875	4
AAGlyITA	89,125	35,625	15	3,625	0	0	917,625	519,375	289,75	149,125	52,875	4,875
AAMEGITA	0	0	0	0	0	0	892,75	462,25	240,375	125,375	46,375	5,125
AAPDOITA	0	0	0	0	0	0	783	423,5	229,25	118,875	46,5	4,75

D. Objects	IW1	IW2	IW3	IW4	CW1	CW2	CW3	CW4	CW5	CW6	CW7	CW8	ID1	ID2	ID3	ID4
AA	0,028953	0,044123	0,079963	0,191286	3,31233	2,80931	1,87816	0,981806	0,542217	0,304805	0,138748	0,025547	0,061851	0	0	0
BDO	0,009403	0,010291	0,055632	0,239428	3,25596	2,76806	1,63696	0,768358	0,412573	0,196436	0,031291	0	0,006603	0,288012	0	0
GLY	0,145819	0,252248	0,530224	1,53496	3,71302	3,28775	2,03586	1,04572	0,574565	0,291483	0,095007	0,019389	0,214595	0,395835	0	0
ITA	0,241963	0,435176	0,856047	1,33254	3,37883	2,86719	1,92092	1,05265	0,621056	0,345481	0,172234	0,043059	0,142234	0,348244	0	0
MEG	0,010365	0,018968	0,020637	0,260773	3,82113	3,51963	2,09477	1,01314	0,532156	0,247972	0,02834	0	0	0	0	0
PDO	0,149261	0,274043	0,476747	0,931672	3,531	3,07108	1,84035	0,910398	0,486894	0,227937	0,035067	0	0,205245	0,328505	0	0
BDOITA	0,119629	0,239363	0,592144	1,58948	2,75537	2,19418	1,40298	0,724559	0,395773	0,212241	0,082367	0,018114	0,064637	0,141982	0,202746	0
GLYITA	0,080795	0,149174	0,32479	0,731865	3,28288	2,86192	1,92417	1,00632	0,559925	0,300421	0,123486	0,021011	0,10148	0,273688	0	0
MEGITA	0,053634	0,107891	0,320901	1,04868	3,35059	2,88768	1,79637	0,853358	0,442389	0,218681	0,065772	0,012987	0,070597	0,201256	0,396558	0
PDOITA	0,097597	0,182774	0,45237	1,25918	3,2143	2,7645	1,84454	0,909583	0,480164	0,251039	0,097648	0,011918	0,108062	0,244243	0	0
AABDOITA	0,082179	0,166662	0,398768	0,5875	2,66294	2,10561	1,39963	0,714198	0,390663	0,217136	0,100006	0,018723	0,063998	0,138865	0,232759	0
AAGlyITA	0,035667	0,068762	0,150864	0,287388	2,82345	2,38663	1,68772	0,946728	0,538702	0,293838	0,131169	0,026578	0,02638	0,098319	0	0
AAMEGITA	0,011678	0,035425	0,101377	0,183626	2,94697	2,47436	1,673	0,85407	0,447661	0,23478	0,112043	0,021645	0,034054	0,029153	0,200102	0
AAPDOITA	0,009166	0,017264	0,065776	0,203176	2,56471	2,05394	1,43854	0,767519	0,430283	0,238773	0,113971	0,024123	0,022612	0,076626	0	0

E.Object	CD1	CD2	CD3	CD4	CD5	CD6	CD7	CD8
AA	0,011893	0	0	0	0	0	0	0
BDO	0,048674	0,015645	0,001159	0	0	0	0	0
GLY	0,040717	0,014865	0,002585	0	0	0	0	0
ITA	0,082571	0,007092	0,000507	0	0	0	0	0
MEG	0	0	0	0	0	0	0	0
PDO	0,042485	0,013487	0,000674	0	0	0	0	0
BDOITA	0,141836	0,041013	0,010253	0,001709	0,000684	0	0	0
GLYITA	0,043128	0,004792	0,001843	0	0	0	0	0
MEGITA	0,077083	0,029744	0,014244	0,004608	0,000419	0	0	0
PDOITA	0,063048	0,016915	0,004229	0,000384	0	0	0	0
AABDOITA	0,125578	0,040642	0,007763	0,00137	0,000457	0	0	0
AAGlyITA	0,098684	0,023133	0,004922	0,00123	0	0	0	0
AAMEGITA	0,061623	0,019608	0,010186	0,004074	0,000255	0	0	0
AAPDOITA	0,111509	0,033477	0,008123	0,002462	0	0	0	0

F.Object	HL1	HL2	A	CP	POL	MW	FLEX	FLEX_RB	NCC	DIFF
AA	307,75	173	0	0	12,016	144,125	1,59015	0,318031	2	0,875158
BDO	178	84,75	3,57621	0,002883	9,388	90,121	0,945874	0,189175	0	1,05023
GLY	222,25	112,75	4,71949	0,004629	8,19	92,0938	0,465835	0,093167	0	1,0725
ITA	306,5	170,5	6,57399	0,00075	9,089	128,083	0,901936	0,300645	2	0,873454
MEG	169	78,75	0	0	5,718	62,0678	0,198346	0,066115	0	1,26772
PDO	180,5	84,5	4,3395	0,001443	7,553	76,0944	0,528762	0,13219	0	1,10084
BDOITA	38,6	124,2	4,80697	0,038996	17,673	201,196	2,14967	0,238852	1	0,703778
GLYITA	303,8	203,75	6,31682	0,002909	16,475	203,169	1,99262	0,221403	1	0,72886
MEGITA	31,0588	47,4545	3,19663	0,043304	15,219	174,151	1,5947	0,199338	0	0,833985
PDOITA	113,545	163,25	5,91072	0,008491	15,838	187,17	1,88558	0,235697	1	0,75316
AABDOITA	50,3235	158,5	6,43112	0,02276	28,885	328,314	4,20071	0,280047	2	0,345092
AAGlyITA	109,45	238,8	3,50877	0,016866	27,687	330,287	3,76615	0,251077	2	0,420708
AAMEGITA	43,95	57,625	1,20566	0,14747	25,215	300,261	3,77494	0,29038	2	0,482578
AAPDOITA	52,9697	97	2,44829	0,042727	27,05	314,288	4,08263	0,291616	2	0,446428

G. Objects	LOGP n-Oct	LOGP c-Hex	PSA	HSA	PSAR	PHSAR	LgD5	LgD6	LgD7	LgD7.5	LgD8	LgD9	LgD10
AA	0,144	-4,774	92,24	191,548	0,325031	0,481549	-0,96469	-2,63876	-4,4266	-4,92536	-5,0668	-5,10315	-5,10573
BDO	-0,102	-4,096	40,46	175,26	0,187558	0,230857	-0,102	-0,102	-0,102	-0,102	-0,102	-0,102	-0,102
GLY	-1,723	-6,471	60,69	132,717	0,313794	0,457288	-1,723	-1,723	-1,723	-1,723	-1,723	-1,723	-1,72304
ITA	-0,495	-4,314	92,24	154,517	0,373809	0,596956	-2,05385	-3,67282	-5,25699	-5,60169	-5,70491	-5,7413	-5,74463
MEG	-1,084	-4,64	40,46	118,328	0,254805	0,341931	-1,084	-1,084	-1,084	-1,084	-1,084	-1,084	-1,08401
PDO	-0,593	-4,368	40,46	144,898	0,21828	0,27923	-0,593	-0,593	-0,593	-0,593	-0,593	-0,593	-0,59301
BDOITA	0,364	-3,919	92,65	273,09	0,253322	0,339266	-0,47598	-1,40832	-2,3352	-2,70711	-2,95048	-3,11345	-3,13369
GLYITA	-1,257	-6,294	112,88	226,228	0,332873	0,498966	-2,09698	-3,02932	-3,9562	-4,32811	-4,57148	-4,73446	-4,75478
MEGITA	-0,618	-4,463	83,83	214,55	0,28095	0,390725	-1,45798	-2,39032	-3,3172	-3,68911	-3,93248	-4,09545	-4,11571
PDOITA	-0,127	-4,191	92,65	232,499	0,284946	0,398496	-0,96698	-1,89932	-2,8262	-3,19811	-3,44148	-3,60445	-3,6247
AABDOITA	1,462	-3,556	144,84	402,627	0,264564	0,359737	-0,03034	-1,85664	-3,46505	-3,72171	-3,77362	-3,78687	-3,78789
AAGlyITA	-0,159	-5,931	165,07	342,864	0,324983	0,481445	-1,65134	-3,47764	-5,08605	-5,34271	-5,39462	-5,40788	-5,4089
AAMEGITA	0,48	-4,1	144,84	346,045	0,295059	0,418559	-1,01234	-2,83864	-4,44705	-4,70371	-4,75562	-4,76887	-4,76989
AAPDOITA	0,971	-3,828	144,84	362,965	0,285227	0,399047	-0,52134	-2,34764	-3,95605	-4,21271	-4,26462	-4,27787	-4,27889

H. Objects	AUS7.4	%FU4	%FU5	%FU6	%FU7	%FU8	%FU9	%FU10
AA	0	59,3021	7,7702	0,16119	0,001777	1,80E-05	1,80E-07	1,80E-09
BDO	2	100	100	100	100	100	99,9999	99,9994
GLY	2	100	100	100	100	99,9999	99,9991	99,9905
ITA	0	30,4717	2,74273	0,061734	0,000701	7,11E-06	7,12E-08	7,12E-10
MEG	2	100	100	100	100	100	99,9999	99,9986
PDO	2	100	100	100	100	100	99,9999	99,9988
BDOITA	0	62,7709	14,4281	1,65812	0,168323	0,016858	0,001686	0,000169
GLYITA	0	62,7709	14,4281	1,65812	0,168323	0,016858	0,001686	0,000169
MEGITA	0	62,7709	14,4281	1,65812	0,168323	0,016858	0,001686	0,000169
PDOITA	0	62,7709	14,4281	1,65812	0,168323	0,016858	0,001686	0,000169
AABDOITA	0	46,5084	3,20861	0,046101	0,00048	4,82E-06	4,82E-08	4,82E-10
AAGlyITA	0	46,5084	3,20861	0,046101	0,00048	4,82E-06	4,82E-08	4,82E-10
AAMEGITA	0	46,5084	3,20861	0,046101	0,00048	4,82E-06	4,82E-08	4,82E-10
AAPDOITA	0	46,5084	3,20861	0,046101	0,00048	4,82E-06	4,82E-08	4,82E-10

I. Objects	DRDRDR	DRDRAC	DRDRDO	DRACAC	DRACDO	DRDODO	ACACAC	ACACDO	ACDODO	DODODO
AA	2,22741	0	0	0	0	0	0	0	0	0
BDO	1,08744	2,12088	2,12088	1,60188	1,60188	1,60188	0	0	0	0
GLY	1,10374	2,60179	2,60179	3,11946	3,11946	3,11946	3,01187	3,01187	3,01187	3,01187
ITA	3,47042	0	0	0	0	0	0	0	0	0
MEG	0	1,02651	1,02651	1,02718	1,02718	1,02718	0	0	0	0
PDO	1,08217	1,0881	1,0881	2,05552	2,05552	2,05552	0	0	0	0
BDOITA	6,50812	9,26096	9,26096	5,46303	5,46303	0	2,29591	2,29591	0	0
GLYITA	5,88468	7,8257	7,05885	6,08128	6,08128	0	2,29888	2,29888	0	0
MEGITA	5,07508	6,8582	6,44923	5,25056	5,25056	0	0,904004	0,904004	0	0
PDOITA	5,77332	7,50261	7,34968	6,16813	6,16813	0	1,26875	1,26875	0	0
AABDOITA	15,8224	10,7823	0	8,60218	0	0	3,9027	0	0	0
AAGlyITA	15,7618	16,9261	8,24925	13,9432	10,6738	0	5,97398	5,58682	0	0
AAMEGITA	13,4008	13,2331	0	7,32612	0	0	2,9835	0	0	0
AAPDOITA	14,3205	11,4064	0	8,20587	0	0	4,18716	0	0	0

J. Objects	SOLY	LgS3	LgS4	LgS5	LgS6	LgS7	LgS7.5	LgS8	LgS9	LgS10	LgS11
AA	1,43924	1,46601	1,66608	2,54794	4,2233	6,10848	6,95919	7,63475	8,30052	8,42387	8,43768
BDO	0,141207	0,141207	0,141207	0,141207	0,141207	0,141207	0,141207	0,141207	0,141207	0,141207	0,141207
GLY	1,68235	1,68235	1,68235	1,68235	1,68235	1,68235	1,68235	1,68235	1,68235	1,68235	1,68235
ITA	2,72475	2,80949	3,24056	4,28364	5,90572	7,65073	8,35833	8,91036	9,55188	9,70428	9,72266
MEG	0,646775	0,646775	0,646775	0,646775	0,646775	0,646775	0,646775	0,646775	0,646775	0,646775	0,646775
PDO	1,11237	1,11237	1,11237	1,11237	1,11237	1,11237	1,11237	1,11237	1,11237	1,11237	1,11237
BDOITA	-0,1988	-0,17379	0,003357	0,641175	1,57351	2,5004	2,87231	3,11568	3,27864	3,29889	3,30097
GLYITA	1,63878	1,6638	1,84094	2,47876	3,4111	4,33798	4,7099	4,95326	5,11624	5,13657	5,13947
MEGITA	-0,06417	-0,03916	0,137985	0,775804	1,70814	2,63503	3,00694	3,2503	3,41327	3,43352	3,43559
PDOITA	0,592167	0,617182	0,794327	1,43215	2,36448	3,29137	3,66328	3,90665	4,06962	4,08986	4,09194
AABDOITA	-0,89523	-0,8553	-0,5629	0,597156	2,42821	4,30332	5,0897	5,63962	6,04463	6,09855	6,10414
AAGlyITA	-0,07981	-0,03987	0,252534	1,41258	3,24364	5,11874	5,90513	6,45505	6,86005	6,91398	6,91957
AAMEGITA	-1,01967	-0,97973	-0,68733	0,472721	2,30377	4,17888	4,96527	5,51518	5,92019	5,97411	5,97971
AAPDOITA	-1,14233	-1,1024	-0,80999	0,350057	2,18111	4,05622	4,8426	5,39252	5,79753	5,85145	5,85704

K. Objects	PB	VD	CACO2	SKIN	LgBB	MetStab	HTSflag	L0LgS	L1LgS	L2LgS	L3LgS	L4LgS
AA	0	-0,87829	-0,9148	-4,38443	-2,00734	100	0	5,4827	4,54987	-0,88859	-1,33601	0,459974
BDO	0	0,192423	0,457053	-4,5684	-0,04856	100	0	0,141207	0	0	0	0
GLY	0	0,269648	-0,20336	-6,23013	-0,35876	100	0	1,68235	0	0	0	0
ITA	0	-0,75542	-1,0228	-4,58896	-1,72435	100	0	6,94077	4,32438	-1,01629	-1,04225	0,418956
MEG	0	-0,00096	0,126371	-5,08544	-0,3295	100	0	0,646775	0	0	0	0
PDO	0	0,2263	0,298957	-4,89818	0,198785	100	0	1,11237	0	0	0	0
BDOITA	0	-0,67414	-0,46726	-3,95395	-1,74385	100	0	1,99755	2,18862	-0,7112	-0,52889	0,352204
GLYITA	0	-0,41984	-1,01987	-5,87464	-2,01818	100	0	3,83518	2,18876	-0,71103	-0,52872	0,35235
MEGITA	0	-0,51621	-0,54615	-4,61144	-1,74388	100	0	2,13218	2,18862	-0,7112	-0,52889	0,352204
PDOITA	0	-0,5764	-0,68985	-4,64695	-1,66953	100	0	2,78852	2,18862	-0,7112	-0,52889	0,352204
AABDOITA	0	-1,1526	-0,96689	-4,0588	-2,26726	100	0	3,39757	4,44364	-1,28355	-1,16004	0,653036
AAGlyITA	0	-1,13535	-1,52043	-5,54428	-2,99033	100	0	4,213	4,44364	-1,28355	-1,16004	0,653035
AAMEGITA	0	-1,26939	-1,28332	-4,35167	-2,51483	100	0	3,27313	4,44364	-1,28355	-1,16004	0,653035
AAPDOITA	0	-1,20166	-1,10743	-4,05617	-2,53842	100	0	3,15047	4,44364	-1,28355	-1,16004	0,653035

L. Objects	DD1	DD2	DD3	DD4	DD5	DD6	DD7	DD8
AA	0,25	0,125	0	0	0	0	0	0
BDO	0,375	0,125	0,375	0	0	0	0	0
GLY	0,25	0,125	0	0	0	0	0	0
ITA	1,375	0,25	0,125	0	0	0	0	0
MEG	0	0	0	0	0	0	0	0
PDO	0,875	0,125	0	0	0	0	0	0
BDOITA	9	6,375	2	0,5	0,5	0	0	0
GLYITA	0,125	0	0	0	0	0	0	0
MEGITA	0,25	0,25	0,125	0	0	0	0	0
PDOITA	2,75	1,5	1,375	0	0	0	0	0
AABDOITA	21	10,125	3,5	0,75	0,125	0,125	0,125	0
AAGlyITA	24,5	14	7,875	1,875	0,5	0,375	0	0
AAMEGITA	3,25	2,5	0,75	0,5	0,875	0,75	0,375	0,25
AAPDOITA	13,875	2,125	4,5	3,625	1,125	0,5	0,125	0

Tables S2A-M-: Molecular descriptors calculated by VolSurf+ for trimers of the investigated monomers. Columns report: size and shape descriptors (V, S, R, G, Flex); hydrophilic and hydrophobic interaction volumes and capacity factors (W1–W8, CW1–CW8, D1–D8, CD1–CD8, DD1–DD8); INTEGY moments (IW1–IW4, ID1–ID4); H-bond donor/acceptor volumes (WO1–WO6, WN1–WN6); mixed descriptors (HL1–HL2, A, CP, POL, DIFF, MW, LOGP, LogD, PSA/HSA); charge state descriptors (NCC, AUS7.4, %FU4–%FU10); pharmacophoric triplets (DRDRDR–DODODO); and ADME-related parameters (SOLY, LgS3–LgS11, L0lgS–L4lgS, CACO2, SKIN, PB, LgBB, MetStab, VD, HTSFlag)

A. Objects	V	S	R	G	W1	W2	W3	W4	W5	W6	W7	W8
NAC_AA_GLY_DMI	1042,88	720,38	1,44767	1,74335	1829	1474,75	1004,88	511,375	293,75	175	85,625	29,125
NAC_AA_BDO_DMI	1101,25	770,702	1,42889	1,82691	1905,88	1511,5	1046,12	517,5	277,25	151	71,875	18,25
Glucose_amine_AA_GLY_DMI	1056,38	749,699	1,40907	1,8272	1939,62	1681,88	1164,38	633,625	376,125	210,875	92,375	24,375
Glucose_amine_AA_BDO_DMI	1066	725,139	1,47006	1,72246	1954,25	1657,62	1124,88	600,875	350	193,25	85,75	24,5
dipept_AA_GLY_DMI	1605,62	1051,43	1,52709	1,99664	2303,62	1665	1074,75	534,125	292,125	154,875	70,75	13,875
dipept_AA_BDO_DMI	1594,88	1005,45	1,58622	1,87971	2201,62	1495,25	951,375	469,125	257,125	137	59,75	13,375
3_LYS_AA_BDO_DMI	1629,88	1099,76	1,48202	2,10411	2489,5	1965	1335,12	698,875	399,125	229,125	113,375	38,25
3_LYS_AA_Gly_DMI	1615,62	1087,72	1,48533	2,0879	2448,25	1922,75	1274,75	656,5	364,25	190,25	80,75	24,375

B. Objects	D1	D2	D3	D4	D5	D6	D7	D8
NAC_AA_GLY_DMI	71	18,375	9,125	4,125	1,375	0,625	0,125	0
NAC_AA_BDO_DMI	90,25	22,25	9,75	4,5	2,75	1,5	0,5	0,375
Glucose_amine_AA_GLY_DMI	46,875	8,625	2,875	0,75	0,125	0	0	0
Glucose_amine_AA_BDO_DMI	69,375	18,375	5,75	1,5	0,375	0	0	0
dipept_AA_GLY_DMI	238,125	98,875	32,5	14,75	8,875	5,625	4	2,375
dipept_AA_BDO_DMI	255,875	92,75	30	10,375	5,875	3	1,125	0,625
3_LYS_AA_BDO_DMI	130,75	46,375	12,875	6,25	3	2,25	1,75	1
3_LYS_AA_Gly_DMI	143,5	56,125	18,5	7,25	3,125	1,875	1,375	0,75

C. Objects	WO1	WO2	WO3	WO4	WO5	WO6
NAC_AA_GLY_DMI	120,25	49,75	23	4,875	0,25	0
NAC_AA_BDO_DMI	32	9,625	3,875	1,375	0,125	0
Glucose_amine_AA_GLY_DMI	409,5	209,375	109,375	47	10,375	2,25
Glucose_amine_AA_BDO_DMI	380,875	185,75	89,125	36,375	8,875	2
dipept_AA_GLY_DMI	297,375	105,125	45,75	16,5	5,25	2,25
dipept_AA_BDO_DMI	229,875	76,125	34,375	14,375	5,375	0,875
3_LYS_AA_BDO_DMI	633,625	308	178,125	106,375	56,75	23,625
3_LYS_AA_Gly_DMI	522,625	232,75	115,75	50,5	13,75	3,5

D. Objects	WN1	WN2	WN3	WN4	WN5	WN6	IW1	IW2	IW3	IW4
NAC_AA_GLY_DMI	1088,75	555,375	304,25	161,875	62,25	8,375	0,033985	0,062109	0,14451	0,220838
NAC_AA_BDO_DMI	1130,88	573	297,875	155	58,875	8,5	0,03697	0,064817	0,171909	0,261164
Glucose_amine_AA_GLY_DMI	1300,38	705,625	397,75	200,375	57,5	3,375	0,021306	0,029114	0,085294	0,110043
Glucose_amine_AA_BDO_DMI	1234,75	653,125	363,75	185	60,625	4,875	0,029731	0,044766	0,083153	0,098679
dipept_AA_GLY_DMI	1193,5	592	304,625	152,5	55,75	4,25	0,052629	0,123269	0,251781	0,363107
dipept_AA_BDO_DMI	1008,25	475,75	247,625	125,375	52	5,75	0,025437	0,065626	0,156811	0,277545
3_LYS_AA_BDO_DMI	1211,5	590,875	310,25	158	64,125	9,375	0,006403	0,017187	0,102424	0,297245
3_LYS_AA_Gly_DMI	1432,38	723	376,25	177,25	53,125	4,5	0,005915	0,018755	0,102395	0,28109

E. Objects	CW1	CW2	CW3	CW4	CW5	CW6	CW7	CW8
NAC_AA_GLY_DMI	2,53894	2,04718	1,39492	0,709868	0,407771	0,242927	0,118861	0,04043
NAC_AA_BDO_DMI	2,47291	1,9612	1,35737	0,671466	0,359737	0,195925	0,093259	0,02368
Glucose_amine_AA_GLY_DMI	2,58721	2,2434	1,55312	0,845173	0,501701	0,28128	0,123216	0,032513
Glucose_amine_AA_BDO_DMI	2,695	2,28594	1,55125	0,828634	0,482666	0,266501	0,118253	0,033787
dipept_AA_GLY_DMI	2,19094	1,58356	1,02218	0,507998	0,277836	0,147299	0,067289	0,013196
dipept_AA_BDO_DMI	2,18968	1,48714	0,946214	0,46658	0,25573	0,136257	0,059426	0,013302
3_LYS_AA_BDO_DMI	2,26367	1,78675	1,21401	0,635478	0,362919	0,20834	0,10309	0,03478
3_LYS_AA_Gly_DMI	2,25081	1,76769	1,17195	0,603555	0,334874	0,174907	0,074238	0,022409

F. Objects	ID1	ID2	ID3	ID4	CD1	CD2	CD3	CD4	CD5	CD6	CD7	CD8
NAC_AA_GLY_DMI	0,053765	0,107306	0,227463	0,166203	0,098559	0,025507	0,012667	0,005726	0,001909	0,000868	0,000174	0
NAC_AA_BDO_DMI	0,025791	0,024722	0,050015	0,102009	0,117101	0,02887	0,012651	0,005839	0,003568	0,001946	0,000649	0,000487
Glucose_amine_AA_GLY_DMI	0,022332	0,130509	0,246663	0	0,062525	0,011505	0,003835	0,001	0,000167	0	0	0
Glucose_amine_AA_BDO_DMI	0,028106	0,07089	0,160027	0	0,095671	0,02534	0,00793	0,002069	0,000517	0	0	0
dipept_AA_GLY_DMI	0,051515	0,104419	0,188814	0,250181	0,226477	0,094039	0,03091	0,014029	0,008441	0,00535	0,003804	0,002259
dipept_AA_BDO_DMI	0,028807	0,126949	0,253819	0,323836	0,254487	0,092247	0,029837	0,010319	0,005843	0,002984	0,001119	0,000622
3_LYS_AA_BDO_DMI	0,025594	0,060555	0,223483	0,325041	0,118889	0,042168	0,011707	0,005683	0,002728	0,002046	0,001591	0,000909
3_LYS_AA_Gly_DMI	0,010081	0,069254	0,140712	0,324416	0,131927	0,051599	0,017008	0,006665	0,002873	0,001724	0,001264	0,00069

G. Objects	HL1	HL2	A	CP	POL	MW	FLEX	FLEX_RB	NCC	DIFF
NAC_AA_GLY_DMI	32,1918	42,4242	4,81594	0,041842	44,484	507,509	4,73667	0,197361	2	0,07479
NAC_AA_BDO_DMI	28,4359	33,5556	1,9651	0,202894	45,682	505,536	5,35847	0,22327	2	0,064244
Glucose_amine_AA_GLY_DMI	130,826	281,167	5,86538	0,007969	45,993	524,493	4,80499	0,192199	1	0,021418
Glucose_amine_AA_BDO_DMI	60,8696	128,833	3,20378	0,030957	47,191	522,52	4,62642	0,185057	1	0,067097
dipept_AA_GLY_DMI	8,98846	10,5	8,42929	0,109275	76,0261	723,85	6,69193	0,180863	2	-0,43208
dipept_AA_BDO_DMI	8,57083	13,2048	8,66498	0,083774	77,2241	721,877	6,29127	0,170034	2	-0,39985
3_LYS_AA_BDO_DMI	31	36,66	2,4835	0,060329	79,2821	747,897	7,17116	0,174906	5	-0,50316
3_LYS_AA_Gly_DMI	19,6892	26,2414	5,00635	0,074531	74,1451	746,889	7,3008	0,169786	0	-0,41415

H. Objects	LOGP n-Oct	LOGP c-Hex	PSA	HSA	PSAR	PHSAR	LgD5	LgD6	LgD7	LgD7.5	LgD8	LgD9	LgD10
NAC_AA_GLY_DMI	-0,973	-8,093	232,24	488,14	0,322385	0,475765	-3,03186	-4,88154	-6,09052	-6,19367	-6,21534	-6,22231	-6,22297
NAC_AA_BDO_DMI	0,648	-5,718	212,01	558,692	0,275087	0,379476	-1,41086	-3,26054	-4,46952	-4,57267	-4,59434	-4,60131	-4,60197
Glucose_amine_AA_GLY_DMI	-3,527	-9	250,73	498,969	0,334441	0,502496	-6,0592	-6,49068	-6,91721	-6,98872	-7,01451	-7,0262	-7,03157
Glucose_amine_AA_BDO_DMI	-1,906	-7,531	230,5	494,639	0,31787	0,465997	-4,4382	-4,86969	-5,29621	-5,36772	-5,39352	-5,4052	-5,41057
dipept_AA_GLY_DMI	2,186	-0,93	236,26	815,171	0,224703	0,289829	-0,22127	-0,12575	-0,11733	-0,12121	-0,13535	-0,28618	-0,79175
dipept_AA_BDO_DMI	3,807	1,445	216,03	789,424	0,214858	0,273655	1,39973	1,49525	1,50367	1,49979	1,48565	1,33482	0,829247
3_LYS_AA_BDO_DMI	-0,235	-7,251	345,2	754,562	0,313886	0,457484	-8,0186	-6,47434	-5,23518	-4,7349	-4,30446	-3,81078	-3,78821
3_LYS_AA_Gly_DMI	-0,235	-7,251	301,79	785,931	0,277452	0,38399	-8,0186	-6,47434	-5,23518	-4,7349	-4,30446	-3,81078	-3,78821

I. Objects	AUS7.4	%FU4	%FU5	%FU6	%FU7	%FU8	%FU9	%FU10
NAC_AA_GLY_DMI	0	22,5402	0,865846	0,010918	0,000112	1,12E-06	1,12E-08	1,12E-10
NAC_AA_BDO_DMI	0	22,5402	0,865846	0,010918	0,000112	1,12E-06	1,12E-08	1,12E-10
Glucose_amine_AA_GLY_DMI	0	0	0	0	0	0	0	0
Glucose_amine_AA_BDO_DMI	0	0	0	0	0	0	0	0
dipept_AA_GLY_DMI	0	0	0	0	0	0	0	0
dipept_AA_BDO_DMI	0	0	0	0	0	0	0	0
3_LYS_AA_BDO_DMI	0	0	0	0	0	0	0	0
3_LYS_AA_Gly_DMI	0	0	0	0	0	0	0	0

J. Objects	DRDRDR	DRDRAC	DRDRDO	DRACAC	DRACDO	DRDODO	ACACAC	ACACDO	ACDODO	DODODO	SOLY
NAC_AA_GLY_DMI	34,7873	33,7074	28,1852	27,0125	23,4325	0	12,8697	17,3351	0	0	-1,80792
NAC_AA_BDO_DMI	32,7196	30,6126	28,0086	26,6598	26,7619	0	13,7292	17,588	0	0	-2,57589
Glucose_amine_AA_GLY_DMI	32,1438	27,9375	0	22,9396	0	0	8,82241	0	0	0	-0,24711
Glucose_amine_AA_BDO_DMI	32,3354	32,7594	0	23,6776	0	0	9,34566	0	0	0	-1,70648
dipept_AA_GLY_DMI	61,086	59,5118	43,8813	52,8799	44,0658	0	32,5845	32,3929	0	0	-5,67329
dipept_AA_BDO_DMI	72,3936	69,952	58,4872	58,2187	53,325	0	31,5529	37,1934	0	0	-6,29308
3_LYS_AA_BDO_DMI	58,9138	58,296	59,8174	52,6569	56,0973	28,1231	28,4496	35,5256	21,3953	6,47615	-5,33214
3_LYS_AA_Gly_DMI	62,49	64,9504	52,7257	57,3908	55,938	24,1164	35,8559	37,6779	25,1183	5,54354	-4,52707

K. Objects	LgS3	LgS4	LgS5	LgS6	LgS7	LgS7.5	LgS8	LgS9	LgS10	LgS11	PB
NAC_AA_GLY_DMI	-1,70637	-1,16122	0,251156	2,11993	3,86544	4,49498	4,88757	5,15405	5,18817	5,19169	0
NAC_AA_BDO_DMI	-2,47434	-1,92919	-0,51681	1,35196	3,09747	3,72701	4,1196	4,38608	4,4202	4,42372	0
Glucose_amine_AA_GLY_DMI	3,07634	2,5719	2,28509	2,71658	3,1431	3,21461	3,24041	3,2521	3,25755	3,29869	0
Glucose_amine_AA_BDO_DMI	1,61697	1,11253	0,82572	1,25721	1,68373	1,75524	1,78104	1,79273	1,79818	1,83932	0
dipept_AA_GLY_DMI	-1,51548	-2,69811	-3,26601	-3,36155	-3,36996	-3,36609	-3,35194	-3,20111	-2,69554	-2,27269	60,7779
dipept_AA_BDO_DMI	-2,13527	-3,3179	-3,8858	-3,98133	-3,98975	-3,98587	-3,97173	-3,8209	-3,31533	-2,89248	73,9785
3_LYS_AA_BDO_DMI	7,31298	5,38738	3,65615	2,05799	0,818039	0,317742	-0,11274	-0,60694	-0,63461	-0,14648	0
3_LYS_AA_Gly_DMI	8,11806	6,19245	4,46123	2,86306	1,62311	1,12282	0,692337	0,198132	0,170467	0,658596	0

L. Objects	VD	CACO2	SKIN	LgBB	MetStab	HTSflag	L0LgS	L1LgS	L2LgS	L3LgS	L4LgS
NAC_AA_GLY_DMI	-1,19877	-1,97178	-6,01443	-3,73574	100	0	2,75801	4,19752	-1,58	-0,8322	0,704107
NAC_AA_BDO_DMI	-1,37136	-1,65652	-5,08623	-3,34236	100	0	1,99004	4,19752	-1,58	-0,8322	0,704107
Glucose_amine_AA_GLY_DMI	-0,68747	-2,37098	-11,5337	-3,98903	100	0	2,95256	0,470413	0,046009	-0,47349	0,357519
Glucose_amine_AA_BDO_DMI	-0,77671	-2,18966	-9,99995	-3,58171	100	0	1,4932	0,470413	0,046009	-0,47349	0,357519
dipept_AA_GLY_DMI	-0,52076	-1,57616	-8,05933	-2,91351	36,223	0	-2,99452	-0,09115	0,993881	-0,20756	0,238813
dipept_AA_BDO_DMI	-0,29043	-1,03436	-6,01761	-2,51179	19,7848	0	-3,6143	-0,09115	0,993881	-0,20756	0,238814
3_LYS_AA_BDO_DMI	-0,29914	-5,16447	-34,5995	-4,63547	100	0	1,74113	-4,01057	1,85622	0,260174	-0,01553
3_LYS_AA_Gly_DMI	-0,05011	-2,65165	-13,6361	-4,5371	96,6686	0	2,54621	-4,01057	1,85622	0,260174	-0,01553

M. Objects	DD1	DD2	DD3	DD4	DD5	DD6	DD7	DD8
NAC_AA_GLY_DMI	18,625	3,75	1,75	1,375	0,875	0,25	0	0
NAC_AA_BDO_DMI	41,25	23,75	12,5	6,125	2,25	1,375	0,125	0
Glucose_amine_AA_GLY_DMI	2,75	3,625	2,125	0,625	0,25	0	0	0
Glucose_amine_AA_BDO_DMI	8	0,625	4,25	2	0,125	0	0	0
dipept_AA_GLY_DMI	106	23,25	16,25	5,875	1,75	1,75	1,25	1,5
dipept_AA_BDO_DMI	53,75	37	24	14,375	7,75	4,75	3,5	1,875
3_LYS_AA_BDO_DMI	45,625	24,25	8,5	2,5	0,125	1	0,875	0,5
3_LYS_AA_Gly_DMI	88,25	41,25	18,375	10,375	4,625	1,25	0,875	0,25

Table S3: Definitions of VolSurf+ descriptors

Size and Shape descriptors	Hydrophilic regions	Hydrophobic regions	INTEGY moments	H-bond donor/acceptor regions	Mixed descriptors	Charge state descriptors	3D Pharmacophoric descriptors	ADME-related descriptors
V: Molecular volume (Å^3), water-excluded volume.<br><br>S: Solvent-accessible surface area (Å^2).<br><br>R: Rugosity, ratio V/S.<br><br>G: Globularity, measure of deviation from spherical shape.<br><br>Flex, Flex_RB: Molecular flexibility, normalized by number of rotatable bonds.	W1–W8: Hydrophilic interaction volumes at different energy thresholds.<br><br>CW1–CW8: Hydrophilic capacity factors (hydrophilic volume per surface unit).	D1–D8: Hydrophobic interaction volumes.<br><br>CD1–CD8: Hydrophobic capacity factors.<br><br>DD1–DD8: Differences in hydrophobic volumes across conformers.	IW1–IW4: Hydrophilic integy moments, unbalance between molecular mass centre and hydrophilic regions.<br><br>ID1–ID4: Hydrophobic integy moments, unbalance between mass centre and hydrophobic regions.	WO1–WO6: H-bond donor volumes.<br><br>WN1–WN6: H-bond acceptor volumes.	HL1–HL2: Hydrophilic–lipophilic balance.<br><br>A: Amphiphilic moment (vector from hydrophobic to hydrophilic domain).<br><br>CP: Critical packing parameter.<br><br>POL: Polarizability.<br><br>DIFF: Diffusivity (Stokes–Einstein).<br><br>MW: Molecular weight.<br><br>LOGP (n-Oct, c-Hex): Octanol/water and cyclohexane/water partition coefficients.<br><br>LogD (LgD5–LgD10): pH-dependent distribution coefficients.<br><br>PSA, HSA: Polar and hydrophobic surface areas.<br><br>PSAR, PHSAR: Ratios of PSA/S and PSA/HSA.	NCC: Number of charged centres.<br><br>AUS7.4: Fraction of uncharged species at pH 7.4.<br><br>%FU4–%FU10: Percentage of unionised species at pH 4–10	DRDRDR, DRDRAC, DRDRDO, DRACAC, DRACDO, DRDODO, ACACAC, ACACDO, ACDODO, DODODO: Areas derived from triplets of pharmacophoric points (Dry, donor, acceptor).	SOLY: Intrinsic solubility.<br><br>LgS3–LgS11: Solubility at different pH values.<br><br>L0lgS–L4lgS: Solubility profile coefficients.<br><br>CACO2: Caco-2 cell permeability (qualitative).<br><br>SKIN: Skin permeability (quantitative).<br><br>PB: Protein binding (%).<br><br>LgBB: Blood–brain barrier distribution.<br><br>MetStab: Metabolic stability with CYP3A4 enzyme.<br><br>VD: Volume of distribution.<br><br>HTSFlag: High-throughput screening promiscuity flag.

.

Additional NMR spectra of enzymatically synthetized PGAI

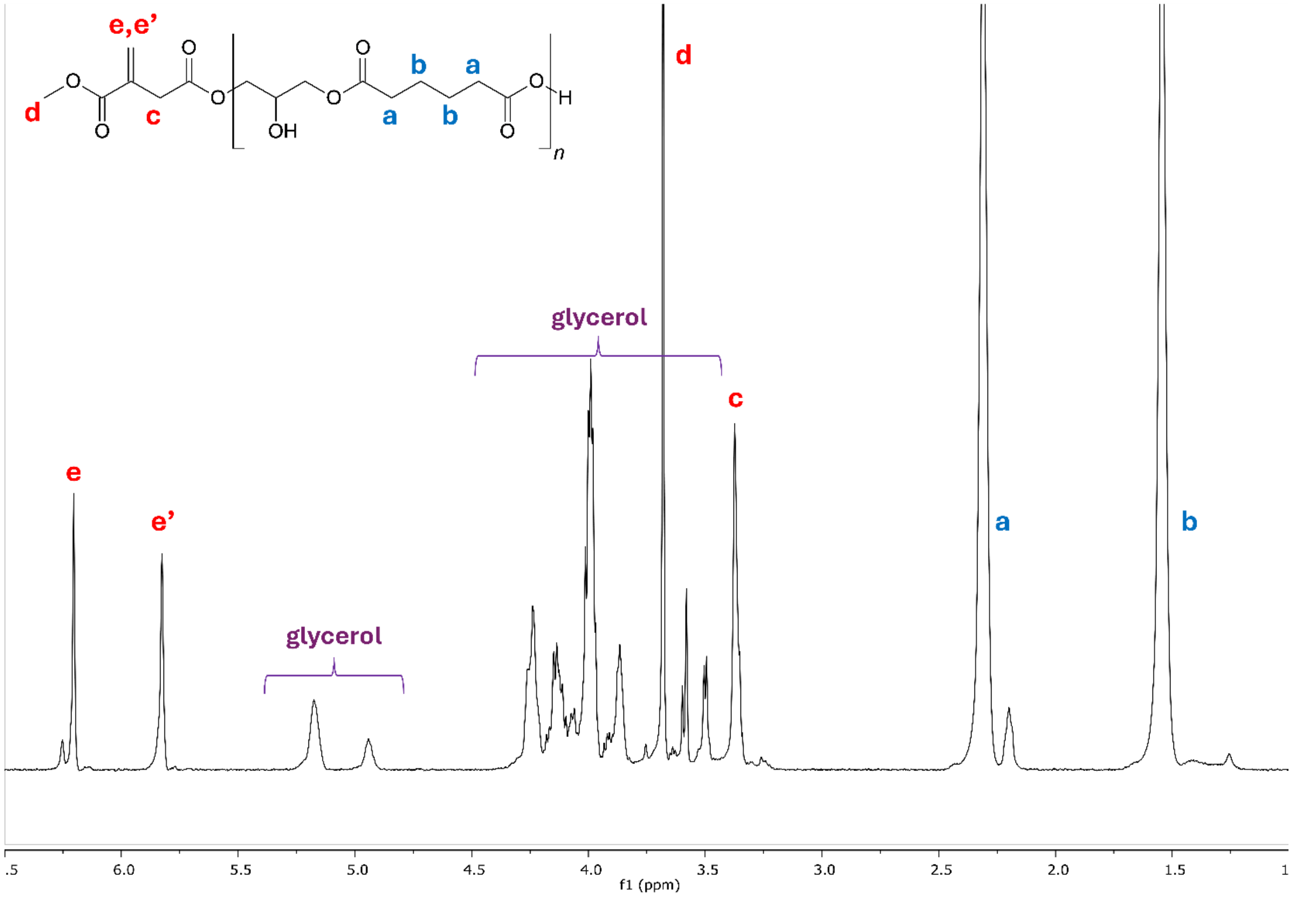

Figure S1: PGSTE ^{1}H NMR spectrum (500 MHz, DMSO-d_6, 45°C, gradient: 60 G cm^{-1}, Δ = 300 ms, δ = 2 ms) of the PGAI terpolymer product, obtained with the highest applied gradient.

Data on marine biodegradability studies on PGAI

Table S4: BOD, ThOD and corresponding biodegration values obtained after 5 and 28 days of incubation of PGAI according to OECD 306 protocols.

Sample	**BOD 5 days** [mg·L^{-1}]	**BOD 28 days** [mg·L^{-1}]	**ThOD** [mg/mg]	**Biodegradability 5 days (%)**	**Biodegradability 28 days (%)**
PGAI	12.15	62.85	41.9	**29%**	**>99%**

Data of the PGAI ecotoxicity study: freshwater organisms

Table S5. Results from the toxicity test performed on *Saccharomyces cerevisiae* exposed 24 h to DMSO (0.5% in ISO freshwater and the oligoester PGAI at a single concentration of 100 mg/L. Data expressed as number of cells per mL of solution (# cell/mL) and percentage of inhibition relative to negative control. NaCl-saturated solution = positive control. Percentage of Inhibition of exposed specimens compared to negative controls; negative values mean increase of cells in tested vessels compared to controls.

Sample	#cell/mL	% inhibition
C-	1.11E+07	0.00
DMSO 0.5%	1.59E+07	-36.68
PGAI	1.35E+07	-18.29
NaCl	5.21E+06	44.91

Table S6. Results of ecotoxicity studies on freshwater organism: test performed on *Pseudokirchneriella subcapitata* exposed for 72 h to different concentrations of PGAI. Legend: C- = negative control, ISO freshwater; DMSO 5% = dimethyl sulfoxide in ISO Freshwater. Data expressed as percentage of inhibition after 72h of exposure compared to negative controls. Mean = mean percentage of Inhibition of exposed specimens compared to negative controls; SD = standard deviation; negative values mean increase of cells in tested vessels compared to controls. *P. subcapitata* (microalgae). The results obtained showed the absence of ecotoxicity for tested microalgae species under tested conditions (0.3-60 mg/L). Compared to DMSO 5% the effect produced by PGAI (60 mg/L) exposure was significantly lower (T-test, $p<0.01$). Results highlighted hormesis under tested concentrations and the absence of ecotoxicological effects.

		**Inhibition (%)**	
	**mg/L**	**Mean**	**SD**
C (-)	0.0	0.0	1.1
DMSO 5%	5%	8.2	7.1
PGAI	0.3	2.8	3.5
	3.0	-0.7	1.9
	30	-1.4	3.4
	60	-10.3	1.2

Table S7. Results of ecotoxicity studies on freshwater organism: test performed on *Daphnia magna* exposed for 24 and 48 h to different concentrations of PGAI. C- = negative control; DMSO= dimethyl sulfoxide 5%. Data expressed as percentage of immobilization after 24h and 48h of exposure. Data is not normalized compared to negative controls. Imm. = percentage of immobilized specimens after 24 and 48 hours of exposure; SD = standard deviation. *D. magna* (Cladocerans). The results obtained showed the absence of ecotoxicity for tested Cladocerans species under tested conditions (0.3-60 mg/L). Compared to DMSO 5% the effect produced by PGAI (60 mg/L) exposure was significantly lower (T-test, $p<0.01$) even if variation associated with recorded responses resulted wider after 48 hours of exposure. Results highlighted the absence of ecotoxicological effects under tested concentrations.

	mg/L	24 hours		48 hours	
		Imm. (%)	SD	Imm. (%)	SD
C-	0.0	10.0	11.5	10.0	11.5
DMSO	5%	10.0	11.5	20.0	0.0
PD5	0.3	0.0	0.0	0.0	0.0
	3.0	0.0	0.0	10.0	11.5
	30	0.0	0.0	10.0	11.5
	60	0.0	0.0	10.0	11.5

Data of the PGAI ecotoxicity study: marine organisms

Table S8. Results of ecotoxicity studies on sea water organisms: test performed on *Aliivibrio fischeri* exposed 15 and 30 minutes to different concentrations of PD5. DMSO= dimethyl sulfoxide 0.5% in sea water. Data expressed as percentage of inhibition of natural bioluminescence after exposure to chemicals normalized by negative control (seawater); negative values mean increase of cells in tested vessels compared to controls. Mean I% = percentage of inhibition of natural bioluminescence after 15 and 30 minutes of exposure; SD = standard deviation. *A. fischeri* (bacteria). The results obtained showed the absence of ecotoxicity for tested bacteria species under tested conditions (0.028-27.9 mg/L). Compared to DMSO 0.5% the effect produced by PD5 (27.9 mg/L) exposure was significantly higher (T-test, $p<0.01$). Results highlighted the absence of ecotoxicological effects under tested concentrations even if at 27.9 mg/L a significant higher effect of exposed compared to controls (I% >10%).

		**15 minutes**		**30 minutes**	
	mg/L	**mean I (%)**	**SD**	**mean I (%)**	**SD**
**DMSO**	0.5 %	-13.68	0.42	-15.85	0.98
**PD5**	0.0279	-6.4	0.35	-9.66	0.11
	0.279	-8.45	0.19	-10.97	1.89
	2.79	-4.01	0.65	-5.28	2.00
	27.9	11.78	0.52	10.11	0.27

Table S9. Results from the toxicity test performed on *Phaeodactylum tricornutum* exposed for 72 h to different concentrations of PD5. C- = negative control, fresh seawater; DMSO= dimethyl sulfoxide 5%. Data expressed as a percentage of inhibition relative to negative controls. Mean = mean percentage of Inhibition of exposed specimens compared to negative controls; SD = standard deviation; negative values mean increase of cells in tested vessels compared to controls. *P. tricornutum* (microalgae). The results obtained showed the absence of ecotoxicity for tested algae species under tested conditions (0.03-31.0 mg/L). Compared to 5%DMSO, the effect produced by PD5 (31 mg/L) exposure was significantly lower (T-test, $p<0.01$). Results highlighted the absence of ecotoxicological effects under tested concentrations even if at 31 mg/L a significant higher effect of exposed specimens compared to controls (I% >10%).

		**Inhibition (%)**	
	mg/L	**Mean**	**SD**
**C-**	0	-2.5	2.1
**DMSO**	5%	36.8	9.9
**PD5**	31	11.7	3.3
	3.1	6.0	0.1
	0.31	3.7	1.5
	0.031	1.7	4.8

Table S10: Results from the toxicity test performed on *Paracentrotus lividus* exposed for 72 h to different concentrations of PD5. C- = negative control, seawater; DMSO= dimethyl sulfoxide 5% in seawater. Data expressed as percentage of abnormal larvae after 72 hours of exposure; data are normalized compared to negative controls according to the Abbott formula. Mean = mean percentage of Inhibition of exposed specimens compared to negative controls; SD = standard deviation; negative values mean increase of cells in tested vessels compared to controls. *P. lividus* (echinoderms). The results obtained showed the absence of ecotoxicity for tested algae species under tested conditions (0.03-31.0 mg/L). Compared to 5%DMSO, the effect produced by PD5 (31 mg/L) exposure was significantly higher (T-test, $p<0.01$). Results highlighted the absence of ecotoxicological effects under tested concentrations at 3.1 mg/L; while at 31 mg/L a significant effect on exposed specimens compared to controls were measured (Abnormal 74%).

	mg/L	Abnormal larvae (%)		
		**Mean**	**SD**	**Abbott**
**C-**	0	6.33	2.08	0,0
**DMSO**	5%	17.33	4.51	11.74
**PD5**	31	75.33	3.06	73.67
	3.1	10.67	2.52	4.63
	0.31	8.67	1.53	2.49
	0.031	6.33	1.15	0.0

Additional NMR spectra of the products obtained *via* Michael addition to PGAI

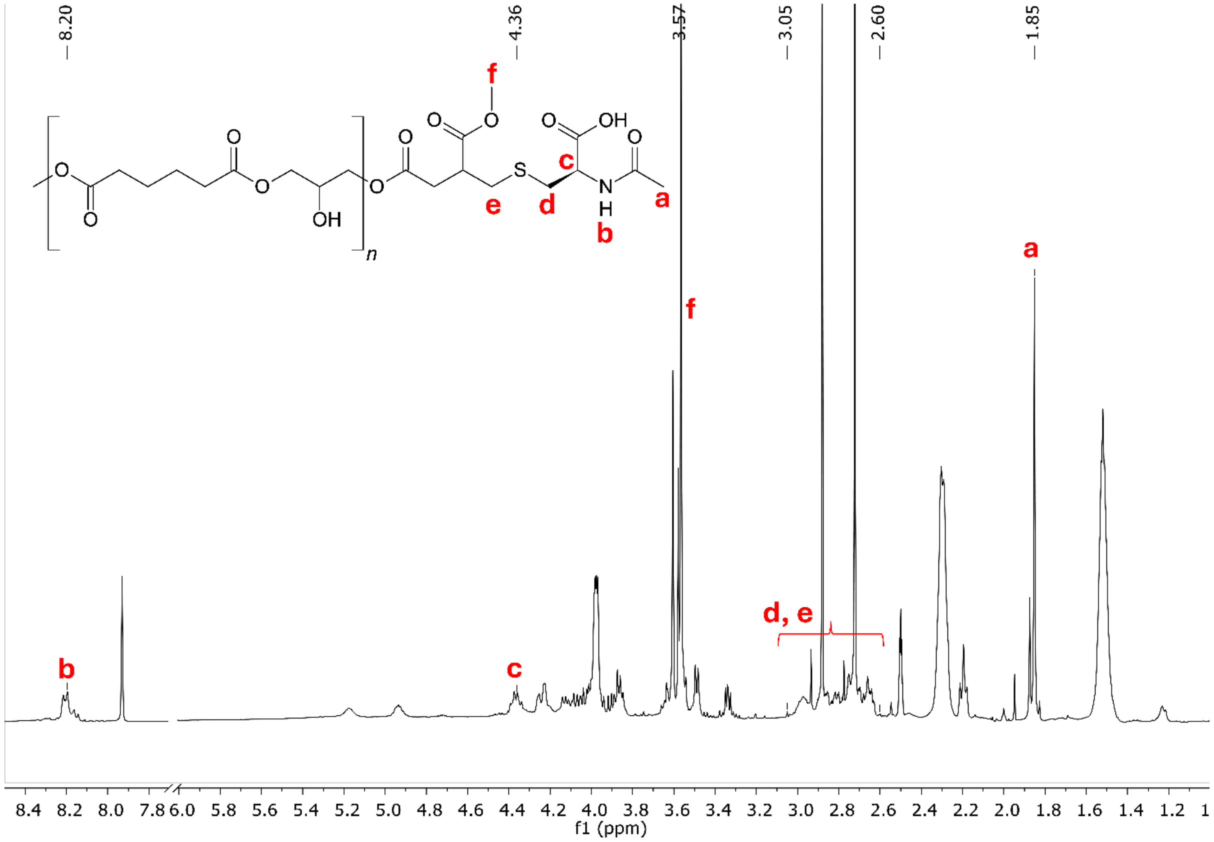

Figure S2: ^{1}H NMR spectrum (400 MHz, DMSO-d$_6$) of the functionalization product of PGAI with NAC. Assigned peaks are characteristic of the product. δ (ppm): 8.20 (N<u>H</u>–CO), 4.36 (Cα cysteine), 3.57 (-CH_3 DMI), 3.05–2.60 (C<u>H$_2$</u>–S– C<u>H$_2$</u>), 1.85 (-CH_3 NAC).

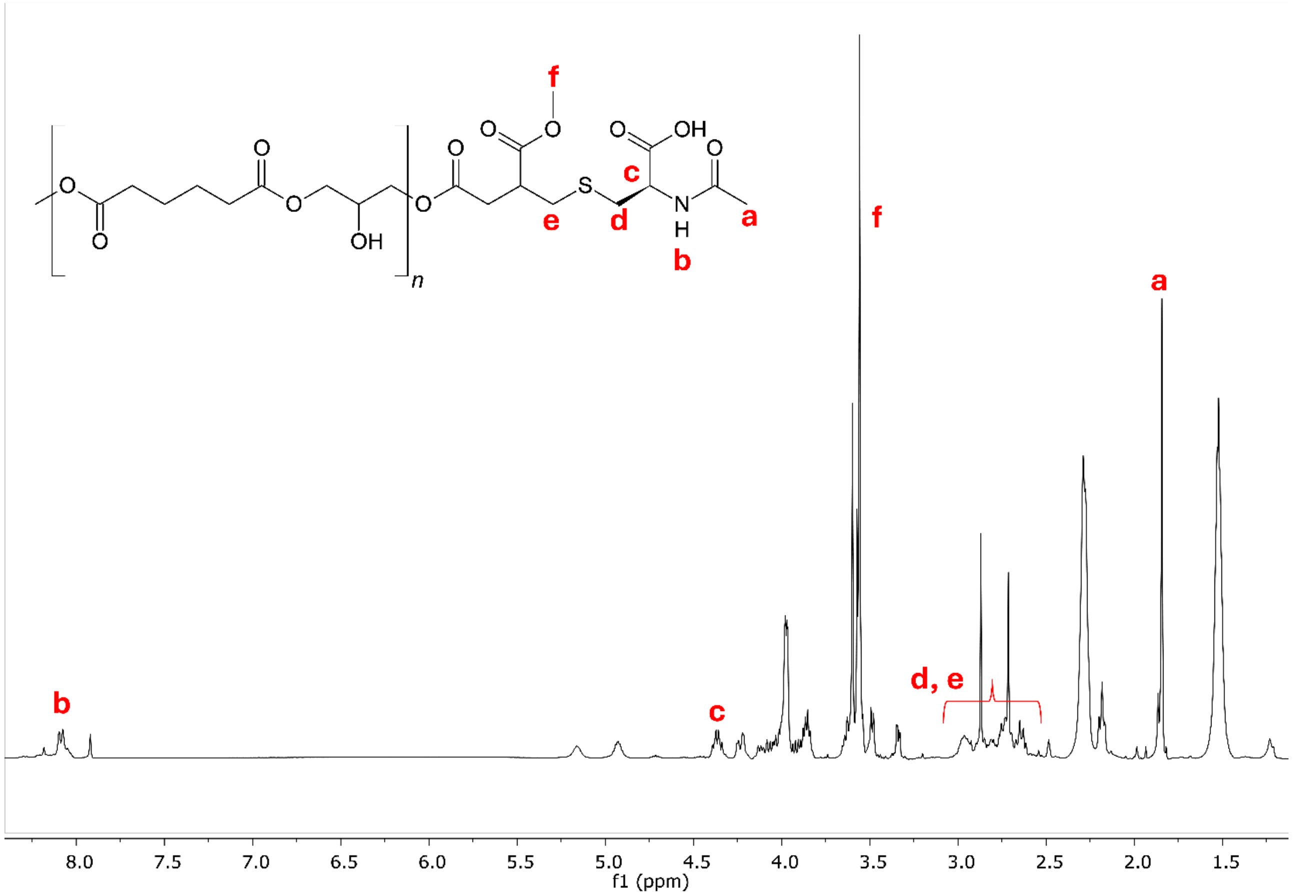

Figure S3: PGSTE ^1H NMR spectrum (400 MHz, DMSO-d_6, 45°C, gradient: 18 G cm^{-1}, Δ = 300 ms, δ = 2 ms) of the PGAI functionalization product with NAC, obtained with the highest applied gradient.

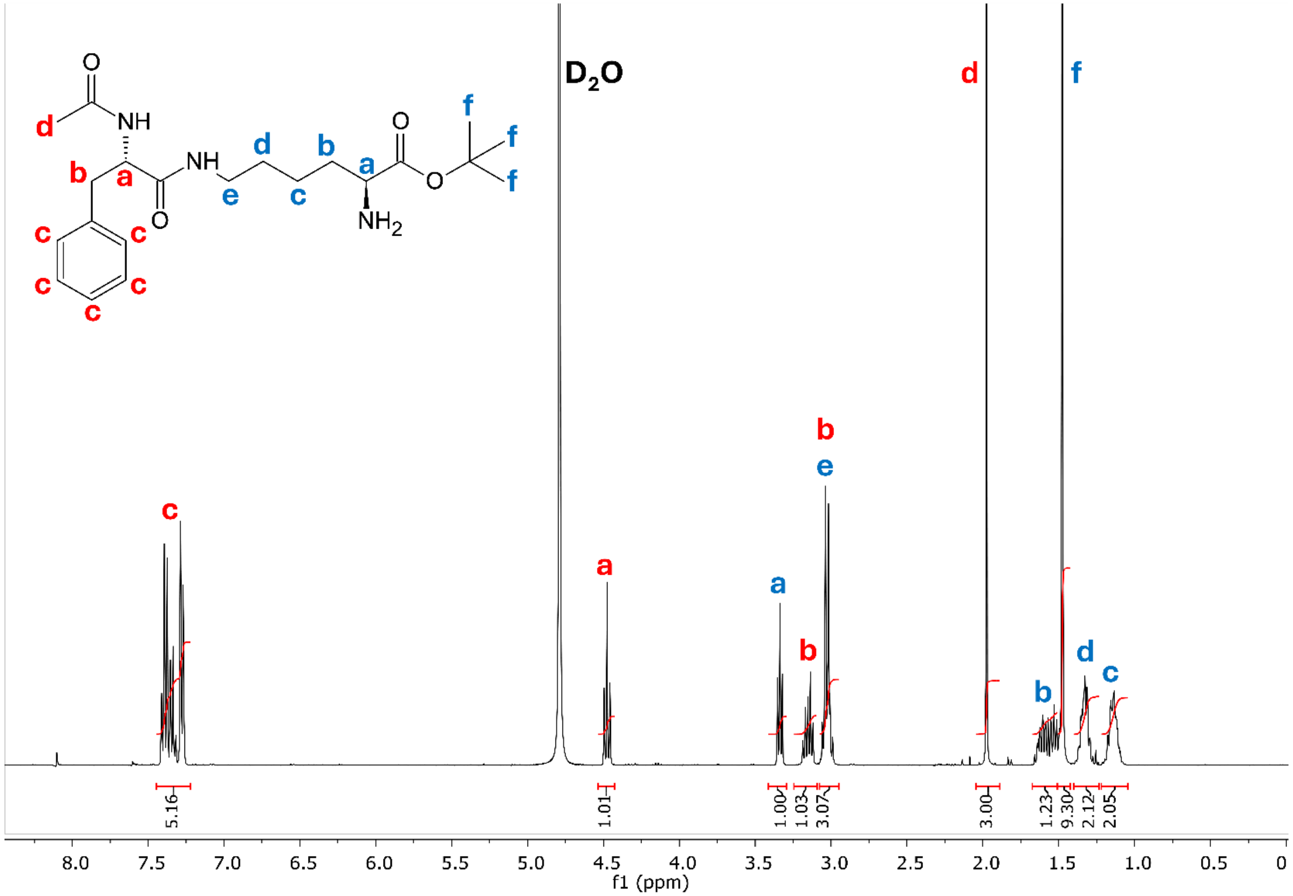

Figure S4: ^{1}H NMR (400 MHz, D_2O) of N-Ac-Phe-ε-Lys-OtBu. In red, signals relative to Phe; in blue, signals relative to Lys. δ 7.45 – 7.22 (m, 5H, Phe aromatics), 4.48 (t, 1H, Cα Phe), 3.34 (t, Cα Lys), 3.15 (m, Cβ Phe), 3.08 – 2.95 (m, Cε Lys and Cβ Phe), 1.98 (s, -CH_3 acetyl), 1.67 – 1.51 (m, Cβ Lys), 1.48 (s, –CH_3 tBu), 1.40 – 1.23 (m, Cδ Lys), 1.14 (m, Cγ Lys). Amide protons are not visible due to exchange with the solvent (D_2O).

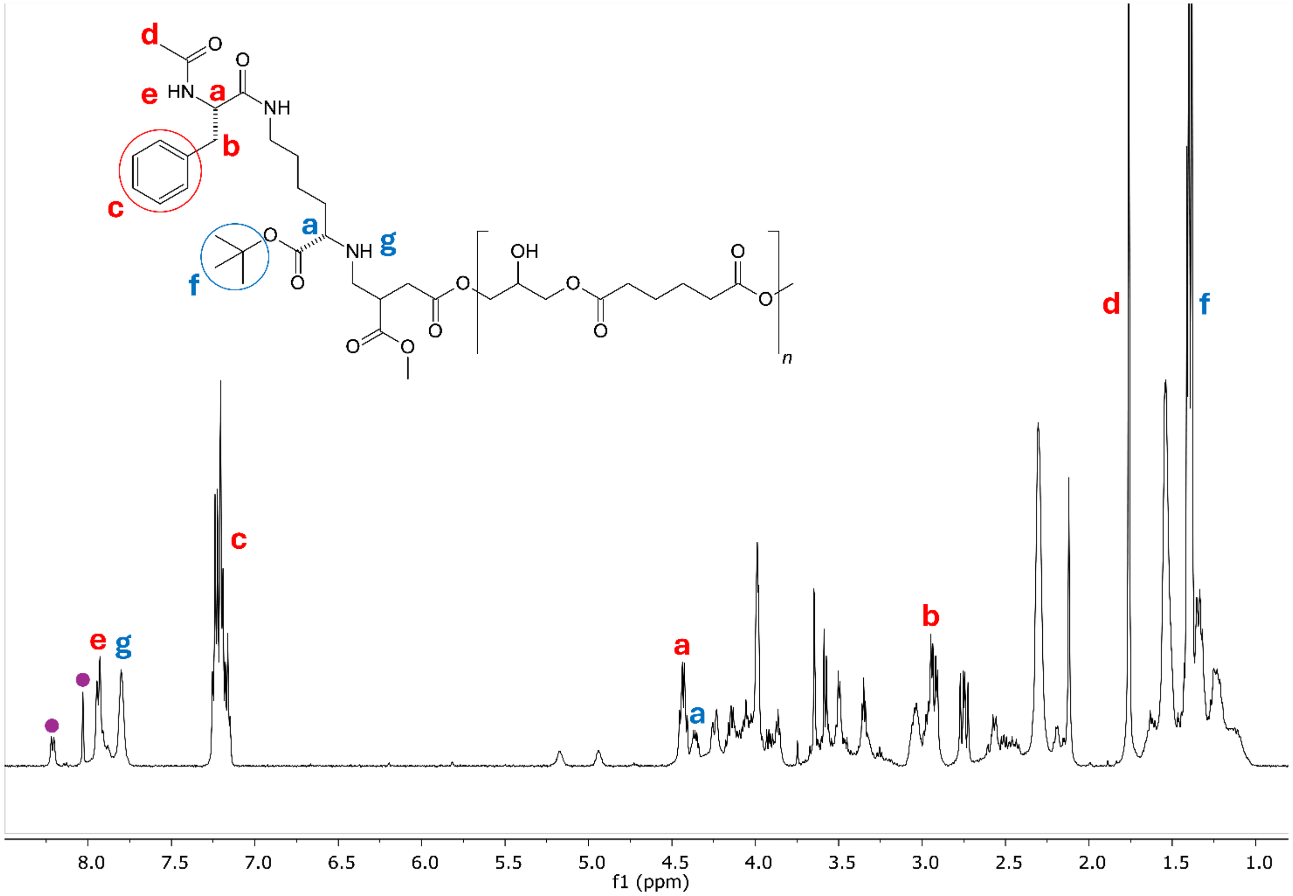

Figure S5: PGSTE ^1H NMR spectrum (500 MHz, DMSO-d_6, 45°C, gradient: 60 G cm^{-1}, Δ = 300 ms, δ = 2 ms) of the reaction product of PGAI with N-Ac-Phe-ε-Lys-OtBu, obtained with the highest applied gradient. In red are assigned signals relative to phenilalanine, in blue signals relative to lysine. Purple dots indicate signals of an unidentified reaction byproduct. Other aminoacid signals are superimposed with peaks relative to the PGAI polymer.

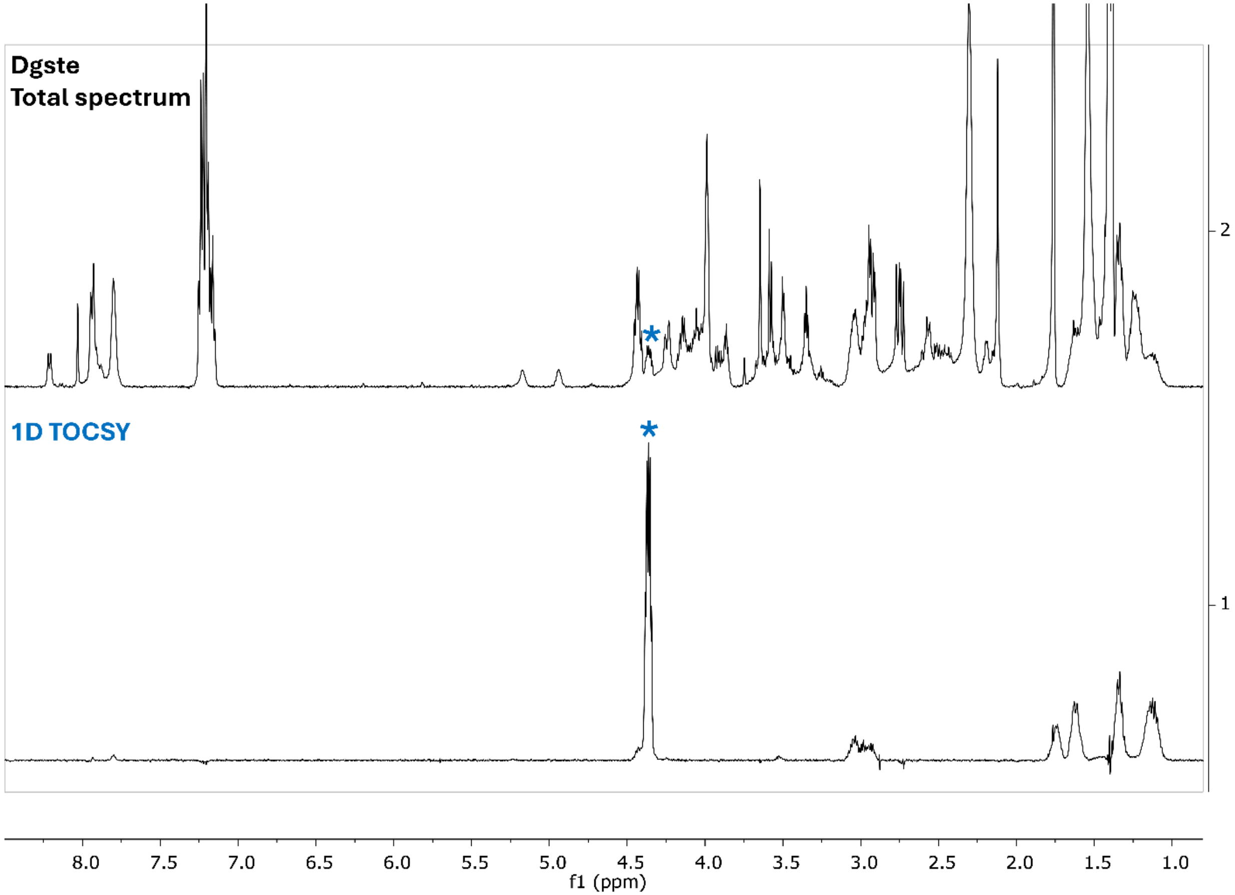

Figure S6: 1D TOCSY NMR spectra (500 MHz, DMSO-d_6, 45°C) of the reaction product of PGAI with N-Ac-Phe-ε-Lys-OtBu. The top spectrum is the entire PGSTE spectrum of the molecule obtained with the highest gradient. The irradiated proton (Cα Lys bound to the polymer) is marked by a blue asterisk.

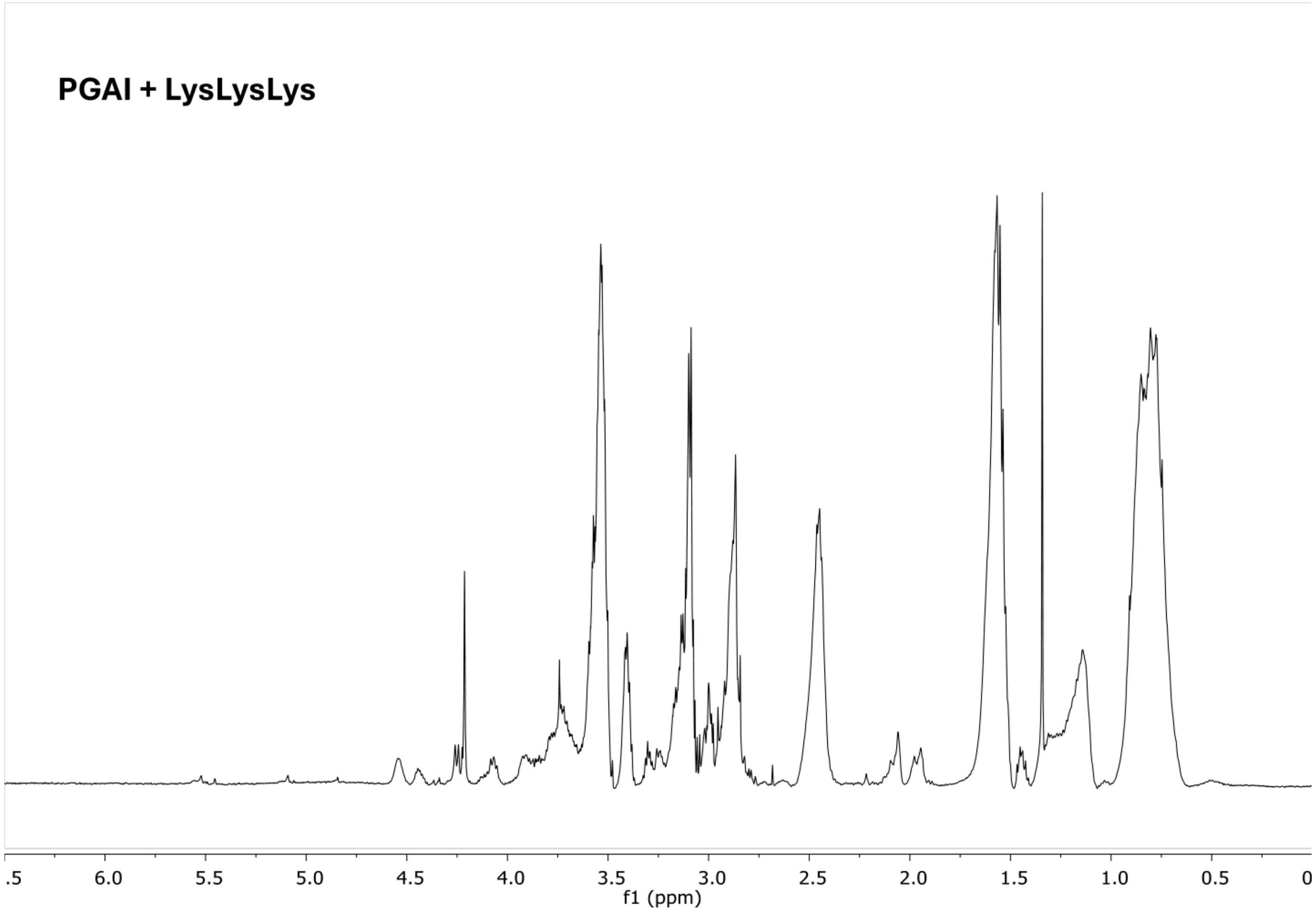

Figure S7: PGSTE ^{1}H NMR spectrum (500 MHz, DMSO-d_6, 45°C, gradient: 60 G cm^{-1}, Δ = 300 ms, δ = 2 ms) of the reaction product of PGAI with N-Ac-Phe-ε-Lys-OtBu, obtained with the highest applied gradient.

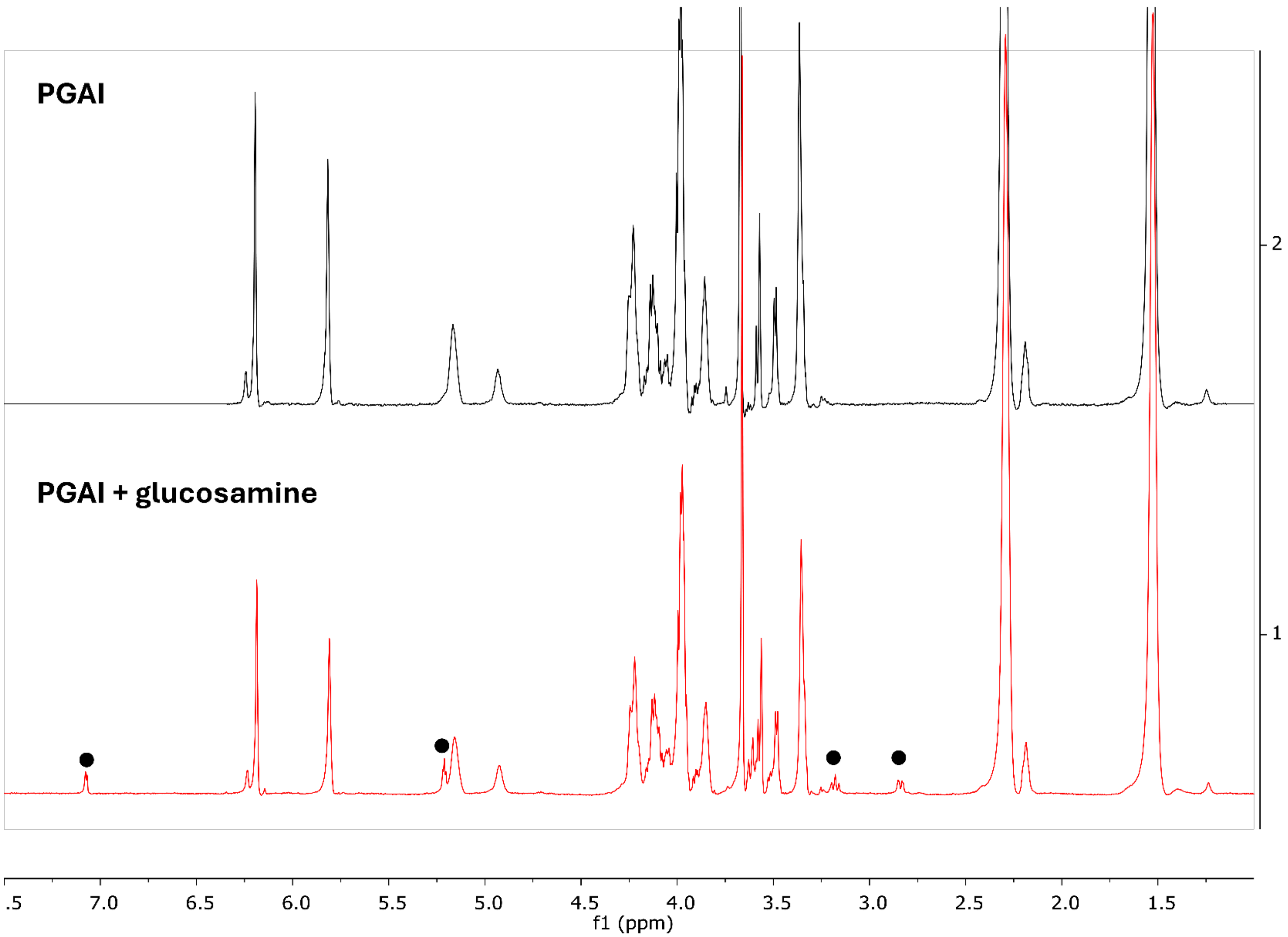

Figure S8: Comparison between the PGSTE ^{1}H NMR spectra (500 MHz, DMSO-d$_6$, 45°C, gradient: 60 G cm^{-1}, Δ = 300 ms, δ = 2 ms) of PGAI and the reaction product of PGAI with glucosamine, obtained with the highest applied gradient. Black dots refer to signals attributed to bound glucosamine.
